# Supplementary material for: The interactome of intact mitochondria by cross-linking mass spectrometry provides evidence for coexisting respiratory supercomplexes
Source: Mol Cell Proteomics. 2017 Dec 8;17(2):216–32. doi: 10.1074/mcp.RA117.000470 (PMC5795388; doi:10.1074/mcp.RA117.000470)
Supplement: Supplemental Data [file supp_RA117.000470_133922_0_supp_23978_fzffwf.zip › spectra_annotation/mito_spectra_annotation/71-1-8-1-4-1.pdf]

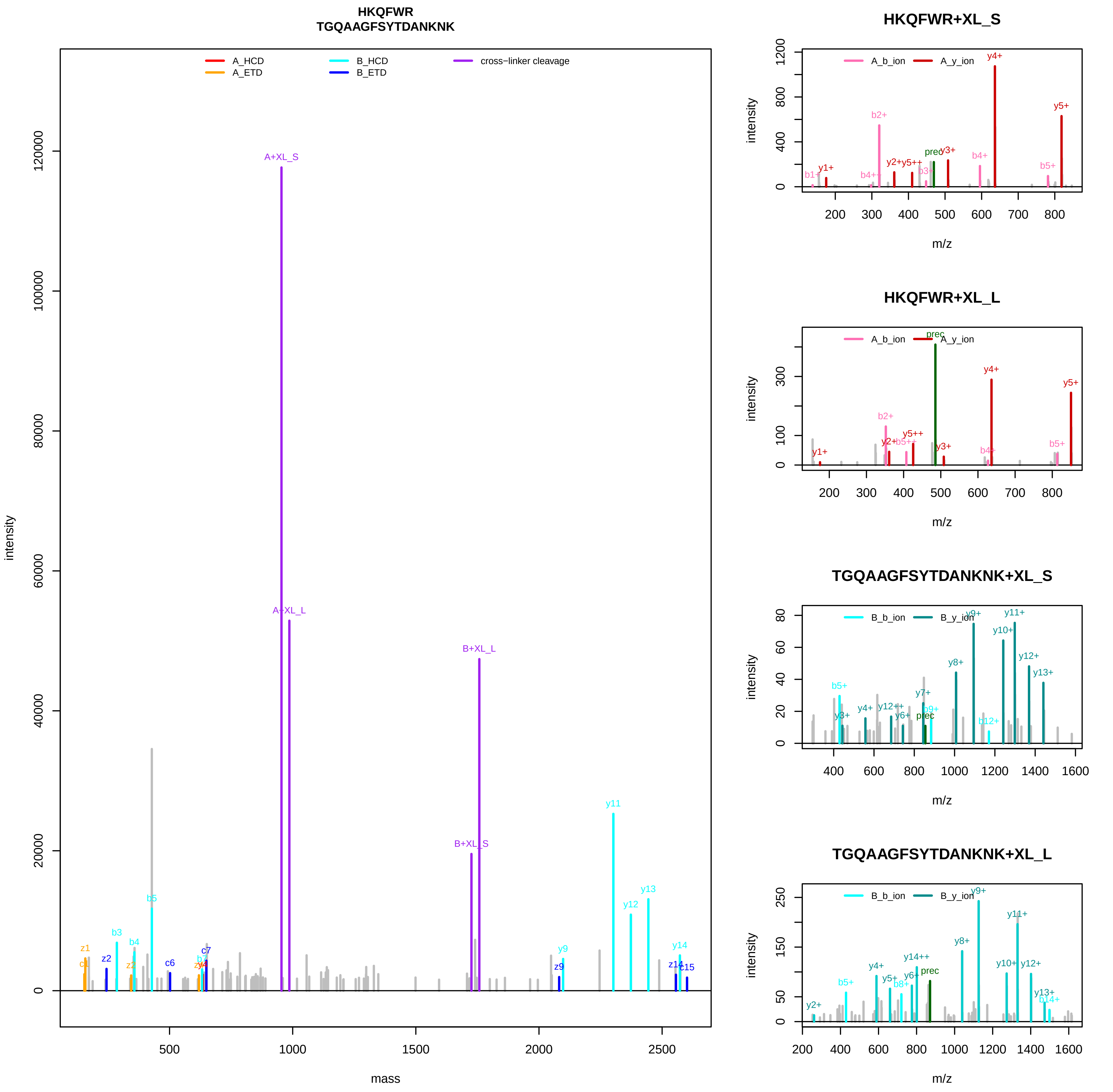
HKQFWR
TGQAAGFSYTDANKNK
A_HCD
A_ETD
B_HCD
B_ETD
cross−linker cleavage
A+XL_S
A+XL_L
B+XL_L
B+XL_S
z1
z2
b3
b4
z2
b5
c6
c7
y4
y9
z9
y11
y12
y13
y14
z14
c15
intensity
mass
0
20000
40000
60000
80000
100000
120000
500
1000
1500
2000
2500
HKQFWR+XL_S
A_b_ion
A_y_ion
b1+
y1+
b2+
b4++
y2+
y5++
b3+
prec
y3+
b4+
y4+
b5+
y5+
intensity
m/z
HKQFWR+XL_L
A_b_ion
A_y_ion
prec
y1+
b2+
y2+
b5++
y5++
y3+
b4+
y4+
b5+
y5+
intensity
m/z
TGQAAGFSYTDANKNK+XL_S
B_b_ion
B_y_ion
b5+
y3+
y4+
y12++
y6+
y7+
b9+
prec
y8+
y9+
y10+
y11+
y12+
y13+
b12+
intensity
m/z
TGQAAGFSYTDANKNK+XL_L
B_b_ion
B_y_ion
y2+
b5+
y4+
y5+
b8+
y6+
y14++
prec
y8+
y9+
y10+
y11+
y12+
y13+
b14+
intensity
m/z
